# NrcR, a New Transcriptional Regulator of *Rhizobium tropici* CIAT 899 Involved in the Legume Root-Nodule Symbiosis

**DOI:** 10.1371/journal.pone.0154029

**Published:** 2016-04-20

**Authors:** Pablo del Cerro, Amanda A. P. Rolla-Santos, Rocío Valderrama-Fernández, Antonio Gil-Serrano, Ramón A. Bellogín, Douglas Fabiano Gomes, Francisco Pérez-Montaño, Manuel Megías, Mariangela Hungría, Francisco Javier Ollero

**Affiliations:** 1 Departamento de Microbiología, Facultad de Biología, Universidad de Sevilla. Sevilla, Spain; 2 Embrapa Soja, Cx. Postal 231, 86001–970, Londrina, Paraná, Brazil; 3 Departamento de Química Orgánica, Facultad de Química, Universidad de Sevilla. Sevilla, Spain; Estacion Experimental del Zaidin—CSIC, SPAIN

## Abstract

The establishment of nitrogen-fixing rhizobium-legume symbioses requires a highly complex cascade of events. In this molecular dialogue the bacterial NodD transcriptional regulators in conjunction with plant inducers, mostly flavonoids, are responsible for the biosynthesis and secretion of Nod factors which are key molecules for successful nodulation. Other transcriptional regulators related to the symbiotic process have been identified in rhizobial genomes, including negative regulators such as NolR. *Rhizobium tropici* CIAT 899 is an important symbiont of common bean (*Phaseolus vulgaris* L.), and its genome encompasses intriguing features such as five copies of *nodD* genes, as well as other possible transcriptional regulators including the NolR protein. Here we describe and characterize a new regulatory gene located in the non-symbiotic plasmid pRtrCIAT899c, that shows homology (46% identity) with the *nolR* gene located in the chromosome of CIAT 899. The mutation of this gene, named *nrcR* (*n**ol**R*-like plasmid c Regulator), enhanced motility and exopolysaccharide production in comparison to the wild-type strain. Interestingly, the number and decoration of Nod Factors produced by this mutant were higher than those detected in the wild-type strain, especially under salinity stress. The *nrcR* mutant showed delayed nodulation and reduced competitiveness with *P*. *vulgaris*, and reduction in nodule number and shoot dry weight in both *P*. *vulgaris* and *Leucaena leucocephala*. Moreover, the mutant exhibited reduced capacity to induce the *nodC* gene in comparison to the wild-type CIAT 899. The finding of a new *nod*-gene regulator located in a non-symbiotic plasmid may reveal the existence of even more complex mechanisms of regulation of nodulation genes in *R*. *tropici* CIAT 899 that may be applicable to other rhizobial species.

## Introduction

The establishment of the symbiosis between rhizobia and their specific host legumes involves highly complex events that culminate in the formation of nodules and in the establishment of the nitrogen fixation process. Nodulation requires a molecular dialogue between the bacteria and the host plants. The dialogue begins with the exudation of molecules from the legume roots, mostly flavonoids, which are recognized by the specific rhizobium, inducing the transcription of a set of nodulation genes [1, 2]. These genes are responsible for the biosynthesis and secretion of Nod factors (NFs), identified as lipochitooligosaccharides, required for launching the nodulation and the nitrogen-fixation process [3–7].

A large number of bacterial transcriptional regulators actuate nodulation, the most important of which are the NodD proteins, which belong to the LysR-type transcriptional-regulator family. The *nodD* genes are constitutively expressed and their cognate proteins are responsible for the recognition of suitable flavonoids, which start the transcription of the nodulation genes [8, 9]. Another important group of transcriptional regulators is the family of metal-sensing regulatory proteins. In this case, a specific metal-ion union regulates the protein’s allosteric conformation and modulates the expression of several target genes. In bacteria, the arsenic repressor family (ArsR) represents one of the most important regulators of the family of metal-sensing proteins. In this family, there are positive and negative transcriptional regulators, which have been described for many bacteria, including several rhizobial species [10–12]. A well-characterized member of this family is the NolR protein, originally described as a general repressor of the activator *nodD1* and the common *nodABC* operon in *Sinorhizobium meliloti* [13–15]. Two models to explain the repression through the NolR protein have been proposed: *i)* in promoters with overlapping transcription initiation and operator sites, NolR binding prevents gene expression enabling RNA polymerase interaction; and *ii)* in promoter regions containing upstream *nod box* (NB) sequences for NodD proteins, NolR binds this promoter region and alters NodD association resulting in inactivation of the gene expression [16]. In *S*. *fredii* HH103, a *nolR* mutant provoked changes in NF decoration and exopolysaccharide production [17]. However, *S*. *meliloti* and *S*. *medicae nolR* mutants increased the *nodC* expression, and, in the case of *S*. *medicae*, improved the competitiveness and nodulation efficiency on *Medicago truncatula* and *Medicago sativa* [18].

*Rhizobium tropici* strain CIAT 899 (hereafter CIAT 899) is a successful microsymbiont of common bean (*P*. *vulgaris* L.) in tropical acid soils. The main features of this broad host-range strain include its high tolerance of environmental stresses such as high temperature, acidity and salinity [19–22]. An intriguing feature of CIAT 899 relies on its capacity of producing a large variety of NFs even in the absence of flavonoids when grown under abiotic stresses such as acid or saline conditions [23–30]. Genome sequencing of strain CIAT 899 displayed a large number of genes that code for transcriptional-regulator families, including five *nodD* genes (all of them located in the symbiotic plasmid; pRtrCIAT899b) and one *nolR* gene (located in the chromosome). Moreover, another gene located in the megaplasmid pRtrCIAT899c and encoding an undescribed protein which shares homology with the canonical NolR protein has also been identified [31]. In this study, we performed experiments to shed light on the role of this new, undescribed *nolR*-like transcriptional regulator gene—which we named *nrcR*—during the symbiotic process.

## Materials and Methods

### Bacteria growth conditions, plasmids, mutant obtention

Strains and plasmids used in this study are listed in Table 1. *R*. *tropici* CIAT 899 strains were grown at 28°C on tryptone yeast (TY) medium [32], B^-^ minimal medium [33] or yeast extract mannitol (YM) medium [34], supplemented when necessary with apigenin 3.7 μM or with NaCl 300 mM. *Escherichia coli* strains were cultured on LB medium [35] at 37°C. When required, the media were supplemented with the appropriate antibiotics as previously described [36]. A similar number of colony forming units (cfu/ml) was obtained for both strains by plating bacteria on addecuated solid medium (data not shown).

**Table 1:** Bacterial strains and plasmids employed in this study.

Strain or plasmid	Derivation and relevant properties	Source or reference
*R*. *tropici* CIAT 899	Wild-type strain, Rif^R^	[19]
*nrcR*:: Ω	*R*. *tropici* CIAT 899 *nrcR*::Ω, Rif^R^ Spc^R^	This work
*nrcR*:: Ω (pMUS1333)	*nrcR* mutant complemented *in trans*, Rif^R^ Spc^R^ Gm^R^	This work
*nrcR*:: Ω (pMUS1353)	*nrcR* mutant complemented *in cis*, Rif^R^ Spc^R^ Km^R^	This work
pMUS1333	pBBR1-MCS-5 containing the entire *nrcR* gene, Gm^R^	This work
pMUS1353	pK18mob containing the entire *nrcR* gene, Km^R^	This work

Primers nolR-like-F (5’–TAG CAG AGC GAT GTC AGA) and nolR-like-R (5’–CGA TGC CAA TTT CCG GAA) were used for amplifying the *nrcR* gene. The 980-bp PCR product was cloned in pGEM®-T Easy (PROMEGA) (Amp^R^ 100 μg mL^-1^) and the resulting plasmid was digested with the enzyme *Hin*dIII, which cuts the *nrcR* gene in one site, and ligated to a 2 kb fragment containg the Ω interposon (Spc^R^ 100 μg mL^-1^) (S1 Fig). Lastly, this plasmid was digested with *Eco*RI and the 2.98-kb fragment containing the *nrcR*::Ω was cloned in the rhizobial suicide vector pK18mob [37], that confers resistance to kanamycin (km^R^ 30 μg mL^-1^), equally restricted with *Eco*RI. The generated plasmid pK18mob containing the *nrcR*::Ω fragment was transferred from *E*. *coli* strain DH5α to *R*. *tropici* CIAT 899 strain by conjugation as described by Simon [38], using plasmid pRK2013 as helper [39], and used for the homogenization of the mutated version of *nrcR* in CIAT 899 by using the methodology previously described [40]. The deletion event was confirmed by PCR and hybridization.

To complement *in trans* the *nrcR* mutation, the pGEM®-T Easy plasmid harboring the 980-bp PCR product that contains the whole *nrcR* gene was digested by *Eco*RI and cloned into the expression vector pBBR1-MCS-5 (Gm^R^ 10 μg mL^-1^) [41], equally digested with the same restriction enzyme, to generate the plasmid pMUS1333. This plasmid was transferred to *ncrR*::*Ω* mutant strain by conjugation, obtaining the *ncrR*::*Ω* (pMUS1333) strain. The *in cis* complementation of the *nrcR* mutant was carried out cloning the *Eco*RI fragment containing the entire *nrcR* gene into the vector pK18mob, generating plasmid pMUS1353. This plasmid was transferred by conjugation into the *ncrR*::Ω strain and the kanamycin resistant transconjugant was selected. The *ncrR*::*Ω* (pMUS1353) strain harbors the plasmid inserted into the pRtrCIAT899c by simple recombination, thus containing both wild-type and mutated *nrcR* gene.

The parental and derivative strains were deposited at the culture collection of the Department of Biology of the Universidad de Sevilla and at the Diazotrophic and Plant Growth Promoting Bacteria Culture Collection of Embrapa Soja (WFCC Collection # 1213, WDCM Collection # 1054).

### Bioinformatics

The Phyre2 web portal (http://www.sbg.bio.ic.ac.uk/phyre2) was used to analyze and predict the function of the NrcR protein [42]. To confirm the prediction obtained, comparisons and similarity analysis among NrcR and the set of protein were carry out using the Basic Local Alignment Search tool (BLAST) suite of NCBI [43].

Specific protein alignments were performed using the ClustalW online platform, [44]. The multiple sequence alignments were edited by using Boxshade at EMBnet.

### Motility assays

Swimming and surface motility phenotypes were assayed on TY and B^-^ minimal medium, supplemented when necessary with NaCl 300 mM or apigenin 3.7 μM. The CIAT 899 and derivative strains were grown in 5 ml of TY medium on an orbital shaker (180 rpm) for 96 h at 28°C. Aliquots of 2 μL of culture suspensions were sink-inoculated in swimming assays (0.28% agar) or drop-inoculated in surface motility assays (0.4% agar) onto Petri dishes and air-dried. Bacterial growth was determined measuring the optical density (O.D.) at 600 nm. All plates were wrapped with parafilm and incubated at 28°C in an upright position and the halo diameters were measure each 24 h. Each experiment was performed three times with three replicates each time.

### Biofilm formation assay

The biofilm formation assay on polystyrene surfaces was performed as previously described [45]. CIAT 899 and derivative strains were grown on TY medium, supplemented with NaCl 300 mM or apigenin 3.7 μM when required, for 7 days with gentle rocking at 28°C. Each experiment was performed three times with eight replicates per time.

### Quantitative and qualitative analyses of external polysaccharides

The anthrone-H_2_SO_4_ method, which measures the total reducing sugar content in a given sample [46], was used to determine the total carbohydrate amounts of EPS contained in supernatants of the bacterial cultures. For this purpose, CIAT 899 and derivative strains were grown in 5 mL of liquid YM medium on an orbital shaker (180 rpm) for 96 h at 28°C. When required, the media were supplemented with NaCl (300 mM) or apigenin (3.7 μM). Samples of 1 mL were centrifuged to remove cells. Cell-free culture supernatants were assayed for EPS content via sulfuric acid hydrolysis in the presence of the colorimetric indicator anthrone. Every experiment was performed three times with three replicates each time.

For the isolation of EPS, CIAT 899 and derivatives strains were cultured for five days at 28°C (late stationary phase) in 45 mL of YM medium and concentrated to about 20% of the initial volume on a rotary evaporator; following, three volumes of cold ethanol were added and the solution was maintained at 4°C for 24 h. Following, the solution was centrifuged and the resulting precipitate was dissolved in water and purified by dialysis against distilled water at 4°C, and then freeze-dried. Monosaccharides were identified on Gas-Liquid Chromatography (GLC Agilent 7809A) coupled with mass spectrometry (MS Agilent 5975 mass detector) separation of their per-O-trimethylsilylated methyl glycosides as previously described [29]. Finally, Nuclear Magnetic Resonance (NMR) was carried out. Samples were deuterium-exchanged twice by freeze-drying from D2O and then examined in solution (around 5 mg 750 μL^-1^) in 99.9% D2O. ^1^H-NMR spectra were recorded at 353 K on a Bruker AV500 spectrometer operating at 500.20 MHz (1H). The HDO signal (4.22 ppm at 353 K) was used as reference.

Lipopolysaccharide (LPS) extraction, separation on SDS-PAGE, and silver staining were performed as previously described using the same bacteria, medium and conditions [29].

### Identification of NFs

Purification and LC-MS/MS analyses of NFs produced by the wild-type and *nrcR* mutant strains growth in B^-^ minimal medium, supplemented when required with NaCl 300 mM or apigenin 3.7 μM, were performed as described previously [27].

### RNA isolation, cDNA synthesis and *q*RT-PCR

The wild-type and *nrcR* mutant strains were pre-cultured in 10 mL aliquots of TY medium at 100 rpm and 28°C in the dark. After 48 h, the strains pre-inoculated were transferred to fresh media supplemented when necessary with 300 mM of NaCl or 3.7 μM of apigenin. These cultures were grown under the same conditions than pre-cultures until to reach an OD 600 nm of 0.5 to 0.6.

Total RNA was extracted using Trizol® reagent (Invitrogen/Life Technologies) as previously described [47]. The total RNA concentration was estimated in a NanoDrop ND 1000 spectrophotometer (NanoDrop-Technologies, Inc.) and the integrity was assessed by gel electrophoresis. Extracted RNA samples were treated with DNAse I (Invitrogen/Life Technologies) and the first stand of cDNA was synthesized using SuperscriptIII^TM^ reverse transcriptase (Invitrogen/Life Technologies), according to manufacturer’s protocol.

Primers for the *q*RT-PCR assays (genes *nodC*, *exoA*, and *exoX*), based on *R*. *tropici* CIAT 899 genome, were designed using Primer3Plus (http://www.bioinformatics.nl/cgi-bin/primer3plus/primer3plus.cgi/), to obtain amplicons of 50–150 bp. A pair of primers for 16S rRNA was also obtained and applied to normalize the relative expression of the targets. To avoid unspecific alignments, the primers sequences were searched against the *R*. *tropici* CIAT 899 genome (Accession numbers NC_020059, NC_020060, NC_020061, NC_020062, for chromosome, pRtrCIAT899a, pRtrCIAT899b and pRtrCIAT899c, respectively).

*q*RT-PCR reactions were performed as described before [29]. The fold changes of three biological samples with three technical replicates of each condition were obtained.

### Nodulation assays

For the evaluation of the symbiotic phenotypes, wild-type and *nrcR* mutant strains of *R*. *tropici* CIAT 899 were grown in YM medium till the concentration of 10^9^ cells mL^-1^. Surface-sterilized seeds of common bean (*P*. *vulgaris*) and leucaena (*L*. *leucocephala*.) were pre-germinated for 2 days at 28°C and placed on sterilized pouch bags or Leonard jars containing Fåhraeus N-free solution [34]. Germinated seeds were then inoculated with 1 mL of bacterial culture or with 1 mL of a mix (1/1, v/v) of two bacterial cultures for competitiveness assays. Growth conditions were 16 h at 26°C in the light and 8 h and 18°C in the dark, with 70% of humidity.

Nodulation parameters were evaluated after 15 days for early nodulation test, and after 30 or 50 days in late nodulation assays for common bean or leucaena, respectively. In all cases, shoots were dried at 70°C for 48 h and weighed.

To evaluate nodule occupancy in the competitiveness assays, 200 nodules from eight plants (25 nodules per plant), were analyzed. Nodules were surface-sterilized [34] and placed on TY plates to confirm lack of surface contamination. These nodules were smashed, streaked in TY with actidione (100 μg/mL), and grown at 28°C for 2–3 days. Isolated colonies were independently picked on TY and TY supplemented with spectinomicyn to discrimine the wild-type and *nrcR*::Ω mutant strain (Spc^R^).

Isolation of the *nrcR*::Ω, *nrcR*::Ω (pMUS1333) or *nrcR*::Ω (pMUS1353) strains from nodules was performed growing nodule extracts on TY plates supplemented with suitable antibiotics.

Nodulation experiments were performed three times.

## Results

### Gene characterization

The sequence of the *R*. *tropici* CIAT 899 genome revealed the presence of an undescribed transcriptional regulator member of the ArsR family (locus tag RTCIAT899_PC05430) located in the megaplasmid pRtrCIAT899c (2.08 Mb) [31]. Protein blast of this transcriptional regulator showed 46% identity and 65% of positive residues with the peptide sequence of the chromosomal *R*. *tropici* CIAT 899 NolR protein (locus tag RTCIAT899_CH13035). Interestingly, the highest homology between the NrcR and NolR proteins was found within the DNA-binding domain (64% of identity and 72% of positive residues), indicating that the proteins share some regulatory DNA targets (S2 Fig). Besides, NrcR also showed high identities with several undescribed ArsR-type proteins (69%–93%) of different soil bacteria and with various rhizobia NolR proteins (41%–48%) (S1 Table). Lastly, the Phyre2 web portal for protein modeling and functioning revealed that this new protein featured the highest values of confidence (99.9) and identity with the crystal structure of *S*. *fredii* NolR protein.

Taking into consideration these data, we decided to name this gene *nrcR*, meaning “**n**ol**R**-like plasmid **c R**egulator.” The *nrcR* gene was mutated by insertion of the Ω interposon and complemented *in cis* and *in trans* with the whole *nrcR* gene copy (See Materials and Methods).

### Studies of the swimming and surface motilities, biofilm formation, EPS production and LPS profiles in the *nrcR* mutant

Phenotypes related to the symbiotic process, such as swimming and on surface motility, biofilm formation, EPS production and LPS profiles, were evaluated in the wild-type and in the *nrcR* mutant strains in the presence or in the absence of apigenin and salt.

No changes were observed in swimming motility, biofilm formation or LPS profile between the wild-type and the *nrcR* mutant strains (data not shown).

**Fig 1:**
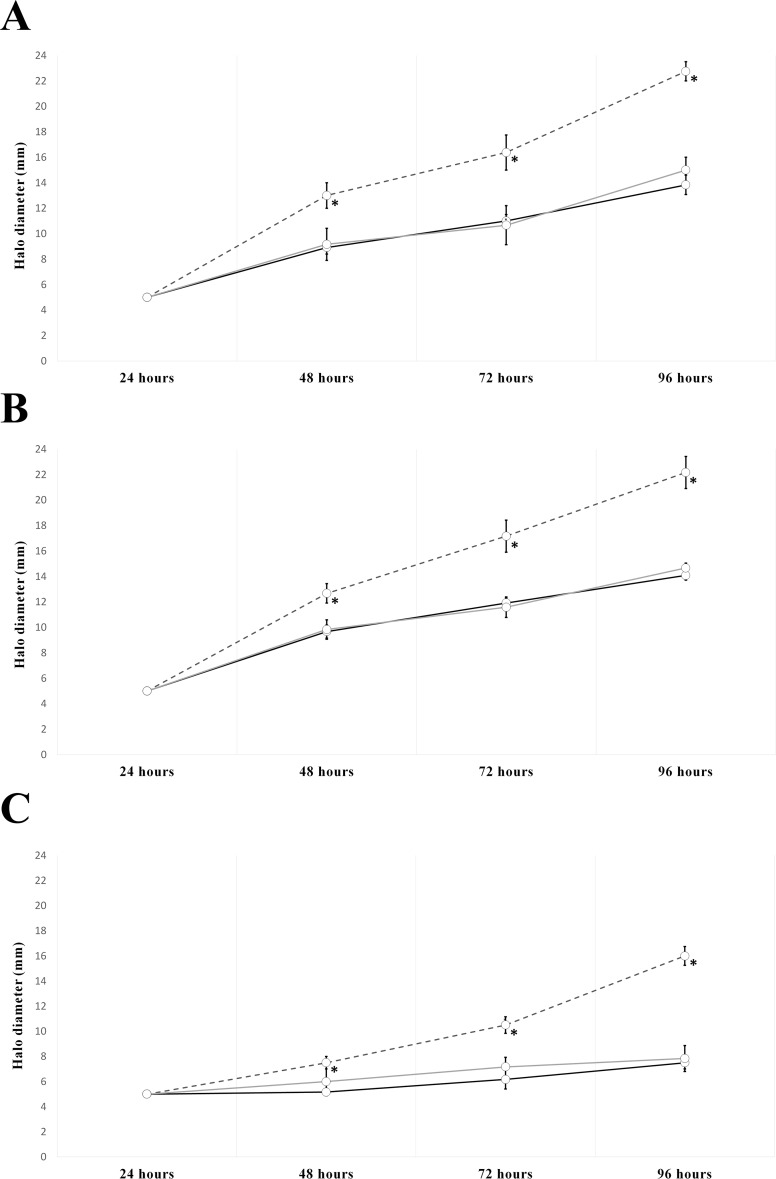
Surface motility phenotype of *R*. *tropici* CIAT 899 and the *nrcR*::Ω mutant strain. Quantified motile ring diameters of wild-type strain (black continuous line), *nrcR*::Ω mutant strain (dark gray discontinuous line) and the *nrcR*::Ω (pMUS1333) complemented strain (light gray continuous line). Values represent the averages of three different experiments per strain. The *nrcR*::Ω and *nrcR*::Ω (pMUS1333) strains parameters were individually compared with their correspondent CIAT 899 strain in each condition by using the Mann-Whitney non-parametric test. Values tagged by asterisks (*) are significantly different (α = 5%). (A) TY medium. (B) TY medium supplemented with 3.7 μM of apigenin. (C) TY medium supplemented with 300 mM of NaCl.

Surface motility was assayed on TY (rich) and B^-^ (minimal) media. Only in the case of the rich-medium assays (TY), were significant differences in surface motility found (Fig 1). The *nrcR*::Ω mutant was about 40% more mobile than the parental strain in TY medium and TY supplemented with apigenin (Fig 1A and 1B). Moreover, in the case of the mutant strain, increased mucosity was observed when bacteria were drop-inoculated on the surface of TY + 0.4% agar medium supplemented or not with apigenin (S3 Fig). The presence of salt resulted in drastic reduction of this motility in all assayed strains. Nevertheless, while the wild-type strain was practically immobile under saline condition, the *nrcR* mutant was mobile (Fig 1C). As expected, these increments in motility were lost in the complemented strain *nrcR*::*Ω* (pMUS1333) in all conditions assayed.

**Fig 2:**
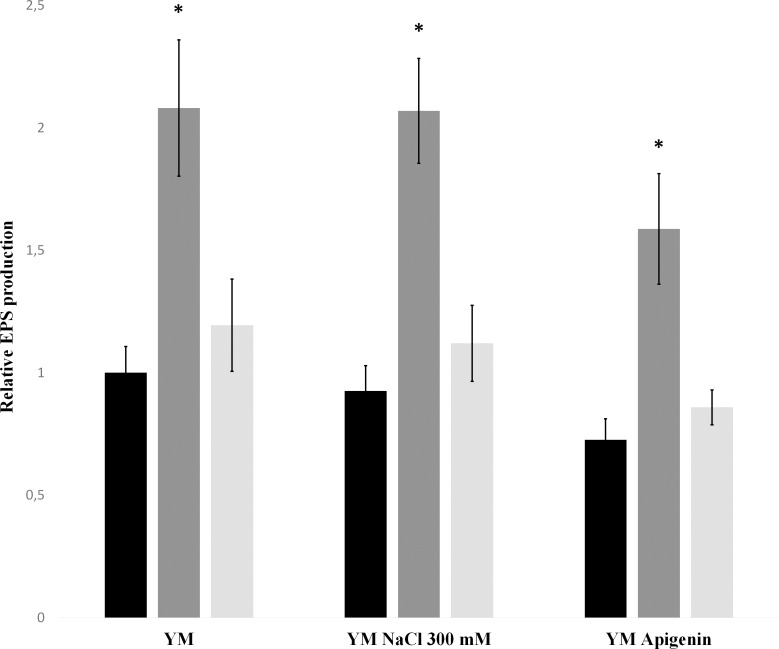
EPS production of *R*. *tropici* CIAT 899 (black bars), the *nrcR*::Ω (dark gray bars) and the *nrcR*::Ω (pMUS1333) (light gray bars) strains. Values were relative to CIAT 899 under control condition. Each experiment was performed three times. Both *nrcR*::Ω and *nrcR*::Ω (pMUS1333) strain parameters were individually compared with the parental strain without induction by using the Mann-Whitney non-parametric test. Values tagged by asterisks (*) are significantly different at the level of α = 5%.

EPS production was evaluated by the anthrone method. The *nrcR*::*Ω* strain produced a significantly higher quantity of EPS than with CIAT 899 strain in all conditions assayed (Fig 2). This phenotype was reversed in the complemented *nrcR*::*Ω* (pMUS1333) strain. Moreover, in the presence of apigenin, the EPS production seemed to slightly decrease in both wild-type and *nrcR* mutant strains (in comparison to the EPS production in YM or YM supplemented with salt). In contrast, *q*RT-PCR experiments were performed to measure the expression of the *exoA* gene, involved in biosynthesis of EPS, and the *exoX* gene, an inhibitor of EPS production, in the mutant and in the wild-type strains. No significant differences in the relative expressions of *exoA* and *exoX* were detected in all assay conditions (data not shown). Finally, we evaluated the EPS composition by monosaccharide analysis and nuclear magnetic resonance (NMR). The sugar composition and 1H-NMR spectra obtained were almost identical in both CIAT 899 and *nrcR*::*Ω* strains (data not shown). Therefore, results suggest that the NrcR transcriptional regulator inhibits, directly or indirectly, the production of EPS, but with no effect on its qualitative composition.

### NF-production patterns and *nod*-gene expression of the *nrcR* mutant

In order to determine if the *nrcR* mutation affects the production and decoration of these molecules, the NF profiles of bacteria grown in B^-^ supplemented or not with apigenin or salt were determined (Tables 2–4) (see Materials and Methods). Under control conditions, both wild-type and *nrcR* mutant strains synthesized 11 and 15 different NFs, respectively. From these NFs, eight were produced by both strains, three were exclusively produced by the parental strain and seven only by the mutant strain (Table 2). Furthermore, in the *nrcR*::*Ω* strain, four NFs presenting five subunits of GlcNAc (V) were sulphated (S), and two of them were also N-methylated (NMe) [V (C_18:1_, S), V (C_18:0_, S), V (C_18:1_, NMe, S) and V (C_18:0_, NMe, S)]. In contrast, the CIAT 899 strain synthesized only one NF sulphated and N-methylated [V (C_18:1_, NMe, S].

**Table 2:** Nod Factor structures biosynthesized under control conditions (B^-^ medium) by the wild-type strain CIAT 899 and the *nrcR*:: Ω mutant.

[M+H]^+^ (*m*/*z*)	B_i_ ions	Structure^a^	CIAT 899^*b*^	*nrcR*:: Ω ^*b*^
**824**	400, 603	III (C_16:0_)	-	+
**838**	414, 617	III (C_16:0_, NMe)	-	+
**850**	426, 629, 832	III (C_18:1_)	+	+
**864**	440, 643	III (C_18:1_, NMe)	-	+
**1027**	400, 603, 806	IV (C_16:0_)	+	+
**1041**	414, 617, 820	IV (C_16:0_, NMe)	-	+
**1053**	426, 629, 832	IV (C_18:1_)	+	+
**1055**	428, 631, 834	IV (C_18:0_)	+	-
**1067**	440, 643, 846	IV (C_18:1_, NMe)	+	+
**1216**	386, 589, 792, 995	V (C_14:0_, NMe)	+	-
**1230**	400, 603, 806, 1009	V (C_16:0_)	+	-
**1244**	414, 617, 820, 1023	V (C_16:0_, NMe)	+	+
**1256**	426, 629, 832, 1035	V (C_18:1_)	+	+
**1270**	440, 643, 846, 1049	V (C_18:1_, NMe)	+	+
**1336**	426, 629, 832, 1035	V (C_18:1_, S)	-	+
**1338**	428, 631, 834, 1037	V (C_18:0_, S)	-	+
**1350**	440, 643, 846, 1049, [M-80]^+^ ^c^ = 1270	V (C_18:1_, NMe, S)	+	+
**1352**	442, 645, 848, 1051, [M-80]^+^^c^ = 1272	V(C_18:0_, NMe, S)	-	+

^a^ NF structures are represented following the convention [64] that indicates the number of GlcNAc residues in the backbone (Roman numeral), the length and degree of unsaturation of the fatty acyl chain, and the other substituents, which are listed in the order in which they appear, moving clockwise from the fatty acid. NMe, *N*-methyl group at glucosamine non reducing residue; S, sulfate group at reducing glucosamine residue.

^b^ Symbol: + = detected;— = non detected.

^c^ These ions arise by loss of a neutral with mass 80 Da, corresponding to the loss of SO_3_.

**Table 3:** Nod Factor structures biosynthesized in the presence of apigenin (3.7 μM) by the wild-type strain CIAT 899 and the *nrcR*:: Ω mutant.

[M+H]^+^ (*m*/*z*)	B_i_ ions	Structure^a^	CIAT 899^b^	*nrcR*:: Ω^b^
**824**	400, 603	III (C_16:0_)	-	+
**838**	414, 617	III (C_16:0_, NMe)	+	+
**850**	426, 629	III (C_18:1_)	+	+
**852**	428, 631	III (C_18:0_)	+	+
**864**	440, 643	III (C_18:1_, NMe)	+	+
**999**	372, 575, 778	IV (C_14:0_)	+	+
**1011**	384, 597, 790	IV (C_14:1_, NMe)	+	-
**1013**	386, 589, 792	IV (C_14:0_, NMe)	+	+
**1025**	398, 601, 804	IV (C_16:1_)	+	-
**1027**	400, 603, 806	IV (C_16:0_)	+	+
**1039**	412, 615, 818	IV (C_16:1_, NMe)	+	+
**1041**	414, 617, 820	IV (C_16:0_, NMe)	+	+
**1053**	426, 629, 832	IV (C_18:1_)	+	+
**1055**	428, 631, 834	IV (C_18:0_)	+	+
**1067**	440, 643, 846	IV (C_18:1_, NMe)	+	+
**1069**	442, 645, 848	IV (C_18:0_, NMe)	+	+
**1081**	454, 657, 860	IV (C_20:1_)	+	+
**1202**	372, 575, 778, 981	V (C_14:0_)	+	-
**1214**	426, 629, 790, 832, 993^d^	V (C_18:1_, dNAc)	+	-
**1216**	386, 589, 792, 995	V (C_14:0_, NMe)	+	+
**1228**	440, 643, 846, 1007^e^	V (C_18:1_, NMe, dNAc)	+	-
**1230**	400, 603, 806, 1009	V (C_16:0_)	+	+
**1242**	412, 615, 818, 1021	V (C_16:1_, NMe)	+	+
**1244**	414, 617, 820, 1023	V (C_16:0_, NMe)	+	+
**1256**	426, 629, 832, 1035	V (C_18:1_)	+	-
**1258**	428, 631, 834, 1037	V (C_18:0_)	-	+
**1270**	440, 643, 846, 1049	V (C_18:1_, NMe)	+	+
**1272**	442, 645, 848, 1051	V (C_18:0_, NMe)	+	+
**1284**	454, 657, 860, 1063	V (C_20:1_)	-	+
**1298**	468, 671, 874, 1077	V (C_20:1_, NMe)	-	+
**1324**	414, 617, 820, 1023	V (C_16:0_, NMe, S)	+	+
**1336**	426, 629, 832, 1035	V (C_18:1_, S)	+	+
**1350**	440, 643, 846, 1049, [M-80]^+^^c^ = 1270	V (C_18:1_, NMe, S)	+	+
**1352**	442, 645, 848, 1051	V (C_18:0_, NMe, S)	-	+
**1378**	468, 671, 874, 1077	V (C_20:1_, NMe, S)	-	+

^a^ NF structures are represented following the convention [64] that indicates the number of GlcNAc residues in the backbone (Roman numeral), the length and degree of unsaturation of the fatty acyl chain, and the other substituents, which are listed in the order in which they appear, moving clockwise from the fatty acid. NMe, *N*-methyl group at glucosamine non reducing residue; S, sulfate group at reducing glucosamine residue.

^b^ Symbol: + = detected;— = non detected.

^c^ These ions arise by loss of a neutral with mass 80 Da, corresponding to the loss of SO_3_.

^d^ Mixture of two Nod Factors, deacetylated at glucosamine residues numbers 2 and 3, respectively.

^e^ Nod Factor deacetylated at glucosamine residue number 2.

**Table 4:** Nod Factor structures biosynthesized in the presence of NaCl 300 mM by the wild-type strain CIAT 899 and the *nrcR*:: Ω mutant.

[M+H]^+^ (*m*/*z*)	B_i_ ions	Structure^a^	CIAT 899^b^	*nrcR*:: Ω^b^
**811**	428, 631	II Hex (C_18:0_)	-	+
**824**	400, 603	III (C_16:0_)	+	+
**838**	414, 617	III (C_16:0_, NMe)	+	+
**850**	426, 629	III (C_18:1_)	+	+
**852**	428, 631, 834	III (C_18:0_)	-	+
**864**	440, 643	III (C_18:1_, NMe)	+	+
**866**	442, 645, 848	III (C_18:0_, NMe)	-	+
**878**	454, 657	III (C_20:1_)	-	+
**892**	468, 671	III (C_20:1_, NMe)	-	+
**999**	372, 575, 778	IV (C_14:0_)	+	+
**1013**	386, 589, 792	IV (C_14:0_, NMe)	+	+
**1025**	398, 601, 804	IV (C_16:1_)	+	-
**1026**	440, 643, 846	III Hex (C_18:1_, NMe)	-	+
**1027**	400, 603, 806	IV (C_16:0_)	+	+
**1028**	442, 645, 848	III Hex (C_18:0_, NMe)	-	+
**1039**	412, 615, 818	IV (C_16:1_, NMe)	-	+
**1041**	414, 617, 820	IV (C_16:0_, NMe)	+	+
**1053**	426, 629, 832	IV (C_18:1_)	+	+
**1055**	428, 631, 834	IV (C_18:0_)	+	+
**1067**	440, 643, 846	IV (C_18:1_, NMe)	+	+
**1069**	442, 645, 848	IV (C_18:0_, NMe)	+	+
**1083**	456, 659, 862	IV (C_20:0_)	-	+
**1095**	468, 671, 874	IV (C_20:1_, NMe)	-	+
**1097**	470, 673, 876	IV (C_20:0_, NMe)	-	+
**1147**	440, 643, 846	IV (C_18:1_, NMe, S)	+	+
**1149**	442, 645, 848	IV (C_18:0_,NMe, S)	+	+
**1177**	470, 673, 876	IV (C_20:0_, NMe, S)	-	+
**1203**	414, 617, 820, 1023	IV Hex (C_16:0_, NMe)	+	+
**1205**	414, 617, 820, 1023	IV Hex-ol (C_16:0_, NMe)	+	-
**1215**	426, 629, 832, 1035	IV Hex (C_18:1_)	+	-
**1216**	386, 589, 792, 995	V (C_14:0_, NMe)	+	+
**1229**	440, 643, 846, 1049	IV Hex (C_18:1_, NMe)	+	+
**1230**	400, 603, 806, 1009	V (C_16:0_)	+	-
**1231**	440, 643, 846, 1049	IV Hex-ol (C_18:1_, NMe)	+	-
**1231**	442, 645, 848, 1051	IV Hex (C_18:0_, NMe)	-	+
**1233**	442, 645, 848, 1051	IV Hex-ol (C_18:0_, NMe)	+	-
**1242**	412, 615, 818, 1021	V (C_16:1_, NMe)	+	+
**1244**	414, 617, 820, 1023	V (C_16:0_, NMe)	+	+
**1256**	426, 629, 832, 1035	V (C_18:1_)	+	+
**1257**	468, 671, 874, 1077	IV Hex (C_20:1_, NMe)	-	+
**1258**	428, 631, 834, 1037	V (C_18:0_)	+	+
**1259**	470, 673, 876, 1079	IV Hex (C_20:0_, NMe)	-	+
**1270**	440, 643, 846, 1049	V (C_18:1_, NMe)	+	+
**1272**	442, 645, 848, 1051	V (C_18:0_, NMe)	+	+
**1284**	454, 657, 860, 1063	V (C_20:1_)	-	+
**1298**	468, 671, 874, 1077	V (C_20:1_, NMe)	+	+
**1300**	470, 673, 876, 1079	V (C_20:0_, NMe)	-	+
**1324**	414, 617, 820, 1023	V (C_16:0_, NMe, S)	+	+
**1336**	426, 629, 832, 1035	V (C_18:1_, S)	+	-
**1350**	440, 643, 846, 1049, [M-80]+^c^ = 1270	V (C_18:1_, NMe, S)	+	+
**1352**	442, 645, 848, 1051	V (C_18:0_, NMe, S)	+	+
**1366**	456, 659, 862, 1065	V (C_20:0_, S)	-	+
**1378**	468, 671, 874, 1077	V (C_20:1_, NMe, S)	+	+
**1380**	470, 673, 876, 1079	V (C_20:0_, NMe, S)	+	+

^a^ NF structures are represented following the convention [64] that indicates the number of GlcNAc residues in the backbone (Roman numeral), the length and degree of unsaturation of the fatty acyl chain, and the other substituents, which are listed in the order in which they appear, moving clockwise from the fatty acid. Hex, hexose; Hex-ol, hexytol (reduced terminal hexose); NMe, *N*-methyl group at glucosamine non reducing residue; S, sulfate group at reducing glucosamine residue.

^b^ Symbol: + = detected;— = non detected.

^c^ These ions arise by loss of a neutral with mass 80 Da, corresponding to the loss of SO_3_.

On the other hand, in the presence of apigenin, both strains synthesized 29 NFs (Table 3), 23 of which were common to both strains, and 6 were exclusive to each strain. Interestingly, only the wild-type strain synthesized NF deacetylated at the glucosamine residue [V (C_18:1_, dNAc) and V (C_18:1_, NMe, dNAc)], while the *nrcR*::*Ω* strain produced NFs with five residues of *N*-acetyl glucosamine and fatty acid of 20 atoms of carbon [V (C_20:1_), V (C_20:1_, NMe) and V (C_20:1_, NMe, S)], which were not synthesized by the parental strain.

Finally, in the presence of salt, the *nrcR* mutant strain synthesized 47 NFs versus 36 produced by the parental strain (Table 4). The CIAT 899 strain produced only 3 NFs with a fatty acid comprising 20 atoms of carbon [V (C_20:0_, NMe), V (C_20:1_, NMe), and V (C_20:0_, NMe, S)], while the *nrcR*::Ω strain synthesized 7 NFs with fatty acids of 20 atoms of carbon, including a backbone with 3, 4, and 5 residues of *N*-acetylglucosamine (Table 4).

In summary, the NFs produced by the *nrcR* mutant strain presented more sulphated substitutions in the reducing *N*-acetylglucosamine residue than those produced by the wild-type strain. In fact, the parental strain produced, in salt conditions, only a sulphated NF, V (C_18:1_, S), which was not produced by the mutant *nrcR*::*Ω* strain (Table 4). Moreover, in relation to the fatty acids of 20 atoms of carbon, the wild-type strain synthesized only 4 of these NFs, one in the presence of apigenin and 3 in salt conditions, while the mutant strain synthesized 15 NFs harbouring C_20_ fatty acid, 4 in the presence of apigenin and 12 in the presence of salt (Tables 3 and 4). Therefore, the study of NF-production patterns indicated that mutation in the *nrcR* gene results in differences in number and decoration of the NFs, especially under salinity stress.

**Fig 3:**
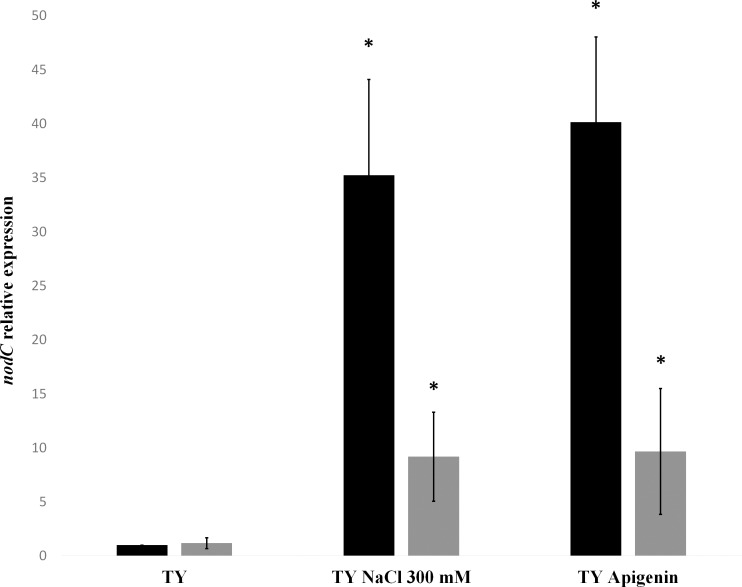
*q*RT-PCR analysis of the expression of *nodC* gene from *R*. *tropici* CIAT 899 and the *nrcR*::Ω strain grown in the absence or presence of apigenin (3.7 μM) or NaCl (300 mM). Expression data were individually compared with the expression without inducing molecules of the wild-type strain by using the Mann-Whitney non-parametrical test. The asterisks (*) indicate a significant different at the level α = 5%. Black bars represent wild-type strain and dark gray bars represent the *nrcR*::Ω mutant strain.

To verify the potential role of the *nrcR* gene in NFs synthesis, the relative expression of *nodC* gene was evaluated. This gene is highly transcribed in the presence both of apigenin and of salt in *R*. *tropici* CIAT 899. In fact, this gene is part of the main set of genes responsible for the synthesis of NFs, the *nodABCSUIJHPQ* operon. qRT-PCR experiments showed that the *nodC* relative expression was significantly lower in the presence both of apigenin and of salt in the *nrcR*::*Ω* mutant strain in comparison with the expression of this gene in the wild-type strain (Fig 3). In the mutant strain, the expression of the *nodC* gene decreased about 35-fold to 9-fold in the presence of salt and about 40-fold to 9.5-fold in the presence of apigenin.

### Symbiotic performance

**Fig 4:**
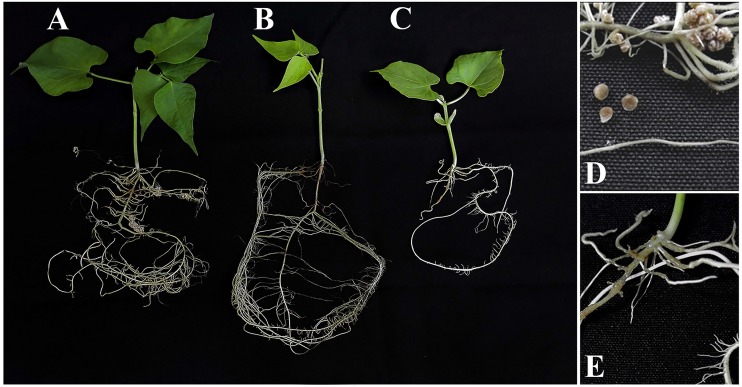
Early nodulation phenotype in common bean inoculated with CIAT 899 and *nrcR*::Ω strains assayed in pouch bags. 1 mL of a culture containg 10^9^ cells mL^-1^ was used as inoculum. Experiment performed under controlled conditions of growth chamber and plants harvested at 15 days after inoculation. (A) wild-type strain. (B) Uninoculated control. (C) *nrcR*::Ω strain. (D) Roots inoculated with the CIAT 899 strain. (E) Roots inoculated with the *nrcR*::Ω strain.

The nodulation phenotype in common bean was firstly evaluated in plants grown in pouch bags for 15 days. Although plants inoculated with CIAT 899 developed nodules 15 days after inoculation, none were observed on plants inoculated with the *nrcR* mutant strain (Fig 4). Furthermore, symbiotic performance was also evaluated in Leonard jars using common bean and leucaena as host plants. At 30 days after inoculation in common bean, the *nrcR* mutant significantly decreased nodule number (by 36%) in comparison to the parental strain. Furthermore, the decrease in nodulation was reflected in lower shoot dry weight (25% less) of plants inoculated with the mutant strain (Table 5).

**Table 5:** Plant responses to inoculation of common bean and leucaena with *R*. *tropici* CIAT 899 and derivative strains.

Strains	*P*. *vulgaris* ^a^		*L*. *leucocephala* ^a^	
	Nodule number	Shoot dry weight (g)	Nodule number	Shoot dry weight (g)
*R*. *tropici* CIAT 899	251.3 ± 30.25	1.58 ± 0.21	17.5 ± 3.1	0.49 ± 0.05
*nrcR*:: Ω	160.2 ± 28.38*	1.18 ± 0.37	10.3 ± 3.73*	0.39 ± 0.03*
*nrcR*:: Ω (pMUS1333)	158.5 ± 36.13*	1.10 ± 0.21*	10.1 ± 3.89*	0.36 ± 0.05*
*nrcR*:: Ω (pMUS1353)	220.6 ± 44.37	1.41 ± 0.33	16.7 ± 3.5	0.48 ± 0.03
none	0 ± 0*	0.70 ± 0.18*	0 ± 0*	0.23 ± 0.05*

^a^ Data represent means ± S.D. of six plant jars. Each jar contained two plants. Common bean plants were evaluated 30 days after inoculation. Leucaena plants were evaluated 50 days after inoculation. The *nrcR*::Ω, *nrcR*::Ω (pMUS1333) and *nrcR*::Ω (pMUS1353) parameters were individually compared with the parental strain CIAT 899 by using the Mann-Whitney non-parametric test. Values tagged by asterisk (*) are significantly different (α = 5%).

When leucaena was inoculated with the *nrcR* mutant, significant differences in nodulation were also observed at 50 days, *i*.*e*. decreases of 41% in nodule number and of 20% in shoot dry weight in comparison to the wild-type strain. The *in trans* complementation [*nrcR*::*Ω* (pMUS1333)] of the *nrcR* mutant did not restore the nodulation phenotypes (Table 5). In fact, the rhizobia isolated from nodules induced by this strain in bean and leucaena plants were Spc^r^ Gm^s^, indicating loss of the pMUS1333 plasmid. For this reason, an *in cis nrcR*-complemented strain [*nrcR*::*Ω* (pMUS1353)] was constructed. In this case, the complementation restored symbiotic characteristics in both plants (Table 5).

**Fig 5:**
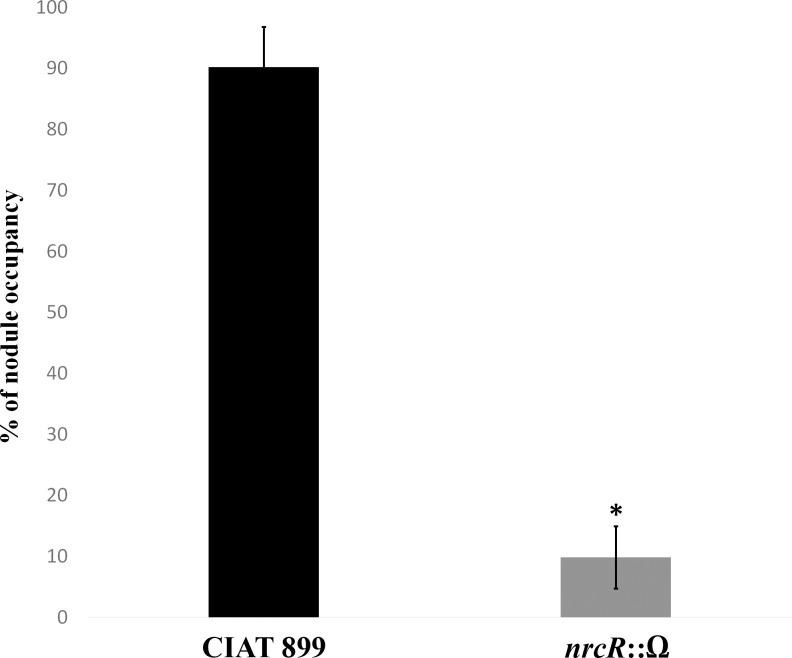
Competitiveness assay of CIAT 899 and *nrcR*::Ω strains in common bean. 0,5 mL of each bacterial culture containing 10^9^ cells mL^-1^ was used as inoculum. Percentages of nodule occupancy were evaluated for both strains in common bean plants after 45 days of inoculation. Values are the averages of three experiments. The *nrcR*::Ω strain values were individually compared with their correspondent wild-type strain CIAT 899 by using the Mann-Whitney non-parametric test. Value tagged by asterisk (*) is significantly different at the level α = 5%.

Moreover, the competitiveness assays showed that the mutant strain was less competitive for nodulation on common bean plants than the wild-type strain (Fig 5). Indeed, after 45 days of inoculation, the *nrcR* mutant occupied only 10% of the nodules.

## Discussion

The participation of NodD transcriptional regulators in conjunction with plant inducers, mostly flavonoids, is required for *nod*-gene expression in rhizobia, which represents the beginning of the nodulation process. The absence of the transcriptional regulator repressor NolR provokes changes in NF decoration as well as decreases in nodulation efficiency in other rhizobial species and strains such as *S*. *fredii* and *S*. *meliloti*, indicating that a fine-tuning of *nod-*gene expression is required for optimal nodulation of the plant host [13, 17]. A structural and biochemical analysis of the NolR protein revealed that a DNA-protein interaction domain is essential to the full understanding of its transcriptional repression role in several target genes [18]. In this study, we investigated a new potential symbiotic regulator belonging to the ArsR family of proteins located in the non-symbiotic plasmid pRtrCIAT899c of *R*. *tropici* CIAT 899. Here, we report that this transcriptional regulator, named NrcR, shares homology in the sequence, especially in the DNA-binding domain, with NolR. We have demonstrated that the mutation of the *nrcR* gene affects several steps of the symbiosis of CIAT 899 with host legumes, revealing a novel level of regulation.

We found that the NrcR transcriptional regulator inhibits surface motility, as the mutant strain consistently showed higher mobility than the parental strain (Fig 1). Nowadays, five types of motility are ascribed to bacteria [48] and it remains to be discovered which one is altered by the absence of NrcR. For this reason, we have generically defined this phenotype as a surface motility type.

The EPS produced by rhizobia has different structural functions, such as a protective barrier against environmental stresses [49, 50]. In CIAT 899, the EPS is an octasaccharide formed by D-glucose, D-galactose, pyruvic and acetic acids, with a ratio of 3:1 between glucose and galactose [51]. Interestingly, the *nrcR*::Ω strain produced a significantly higher amount of EPS than the parental CIAT 899 (Fig 2), but with no difference in the proportion of sugar detected in the NMR analysis. In addition to the protective role of the CIAT 899 EPS, it is worth mentioning that an increase in the EPS production by *S*. *fredii* HH103 promotes surface motility [52]. Thus, it is possible that the over-production of EPS by the *nrcR* mutant could contribute to its increased motility. Remarkably, *nrcR* gene is located in the CIAT 899 megaplasmid that also carries the *exo* genes, which are implied in the biosynthesis of EPS [53]. Thus, the relative position of all these genes is another clue that could be relating EPS production with the NrcR protein. However, the higher production of EPS by the *nrcR* mutant was not related to an increased expression of the *exoA* gene (involved in the biosynthesis of EPS), nor to a repression of the *exoX* gene (a negative regulator of the EPS biosynthesis in rhizobia). Therefore, it is possible that the *nrcR* mutant shows higher capacity of exportation of EPS than the parental strain.

Moreover, a mutation in the *nrcR* gene provoked a clear change in the number and decoration of the NFs synthesized, especially under saline conditions (Tables 2–4). It has been established that CIAT 899 is a host-promiscuous strain that shows high tolerance of environmental stresses [19–22]. Interestingly, the synthesis of a large variety of NFs from *R*. *tropici* CIAT 899 has been related to both features [23–30]. In contrast, *q*RT-PCR experiments showed decreases in the relative expression of the *nodC* gene in the *nrcR* mutant in comparison to that of the parental strain in the presence both of apigenin and of salt (Fig 3). Nevertheless, *nodC* expression in the mutant strain supplemented with apigenin or salt were 9.5-fold and 9-fold respectively higher than in the parental CIAT 899 strain grown without inducers, confirming that the *nrcR* mutant is able to synthesize NFs. Consequently, the *nrcR*::Ω strain could decrease the quantity of NFs but increasing their diversity in comparison to those produced by the parental strain.

The role of the NrcR transcriptional regulator in the symbiosis was also confirmed in nodulation tests with common bean and leucaena. The main effect was observed in the early steps of nodulation, with absence of nodules at 15 days of inoculation with the mutant strain (Fig 4). In addition, when co-inoculated with the wild-type strain, nodule occupancy by the mutant was far lower, possibly related to a lower root-colonization capacity (Fig 5). Competitiveness is critical under field conditions to the success or failure of introduced inoculant strains [54–59]. Furthermore, certain symbiosis-related phenotypes, such as motility or EPS production, can also affect bacterial competitiveness [60, 61]. It has been described that the mutation on the *exoA* gene, decreasing EPS production, increases the competitiveness of *S*. *fredii* HH103 for soybean nodulation [52]. Therefore, we may suppose that decreased competitiveness of the *nrcR* mutant strain is related to the EPS over-production caused by the mutation. Lastly, the *nrcR in trans* complementation [*nrcR*::Ω (pMUS1333)] did not restore the capacity of nodulating bean and leucaena, because the strain lost the pMUS1333 plasmid, as demonstrated after the re-isolation of bacteria from nodules. However, the *in cis* complementation of the mutant strain [*nrcR*::Ω (pMUS1353)] restored the symbiotic phenotype (Table 5).

In summary, NolR is a global regulatory protein that responds to environmental factors and regulates the expression of intracellular metabolic genes [62, 63]. We hypothesize that the high tolerance of *R*. *tropici* CIAT 899 to environmental stresses is coordinated by a complex mechanism involving a broad range of transcriptional regulators. Evolution would have selected some of these regulators, such as the NrcR protein, to regulate nodulation and other features such as EPS production and motility even when *nod*-gene inducing molecules are limited. Therefore, the NrcR transcriptional regulator may represent an important strategy of CIAT 899 to succeed as a symbiont in stressing tropical conditions. In this context, the higher EPS production and motility detected in the *nrcR* mutant may represent an additional protective mechanism for tolerating abiotic stresses. Moreover, symbiotic behavior of the mutant could also represent an indirect effect of these protective mechanisms, affecting probably the early steps of nodulation. The activation of this transcriptional regulator seems to be dependent on flavonoids or salt. Therefore, our results suggest that NrcR is a significant component in the regulatory network present in *R*. *tropici* CIAT 899, which is necessary for orchestration of the symbiotic machinery and other important processes such as tolerance of abiotic stresses.

## Supporting Information

S1 Fig (A) Gene neighborhood of *nrcR* gene in the pRtrCIAT899c of *R*. *tropici* CIAT 899 genome. (B) *Hin*dIII endonuclease point and primers nolR-like-F and nolR-like-R location (C) Mutant interposon insertion.
(TIF)

S2 Fig Alignment of NolR, ArsR and NrcR amino acid sequences using the ClustalW online platform and manipulated with Boxshade at EMBnet. Black and gray boxes indicate identical and similar amino acids, respectively. (A) Complete sequence alignment of NolR of *S*. *fredii* HH103, and NolR, ArsR and NrcR proteins of *R*. *tropici* CIAT 899. (B) Putative DNA-binding domain of NolR of *S*. *fredii* HH103, and NolR and NrcR of *R*. *tropici* CIAT 899.
(TIF)

S3 Fig Mucous halo observed in surface motility of *R*. *tropici* CIAT 899 assays on TY supplemented with 0.4% agar. (A) CIAT 899. (B) *nrcR*::Ω mutant.
(TIF)

S1 Table (PDF)
