# Supplementary figures and images for: NrcR, a New Transcriptional Regulator of Rhizobium tropici CIAT 899 Involved in the Legume Root-Nodule Symbiosis
Source: PLoS One. 2016 Apr 20;11(4):e0154029. doi: 10.1371/journal.pone.0154029 (PMC4838322; doi:10.1371/journal.pone.0154029)

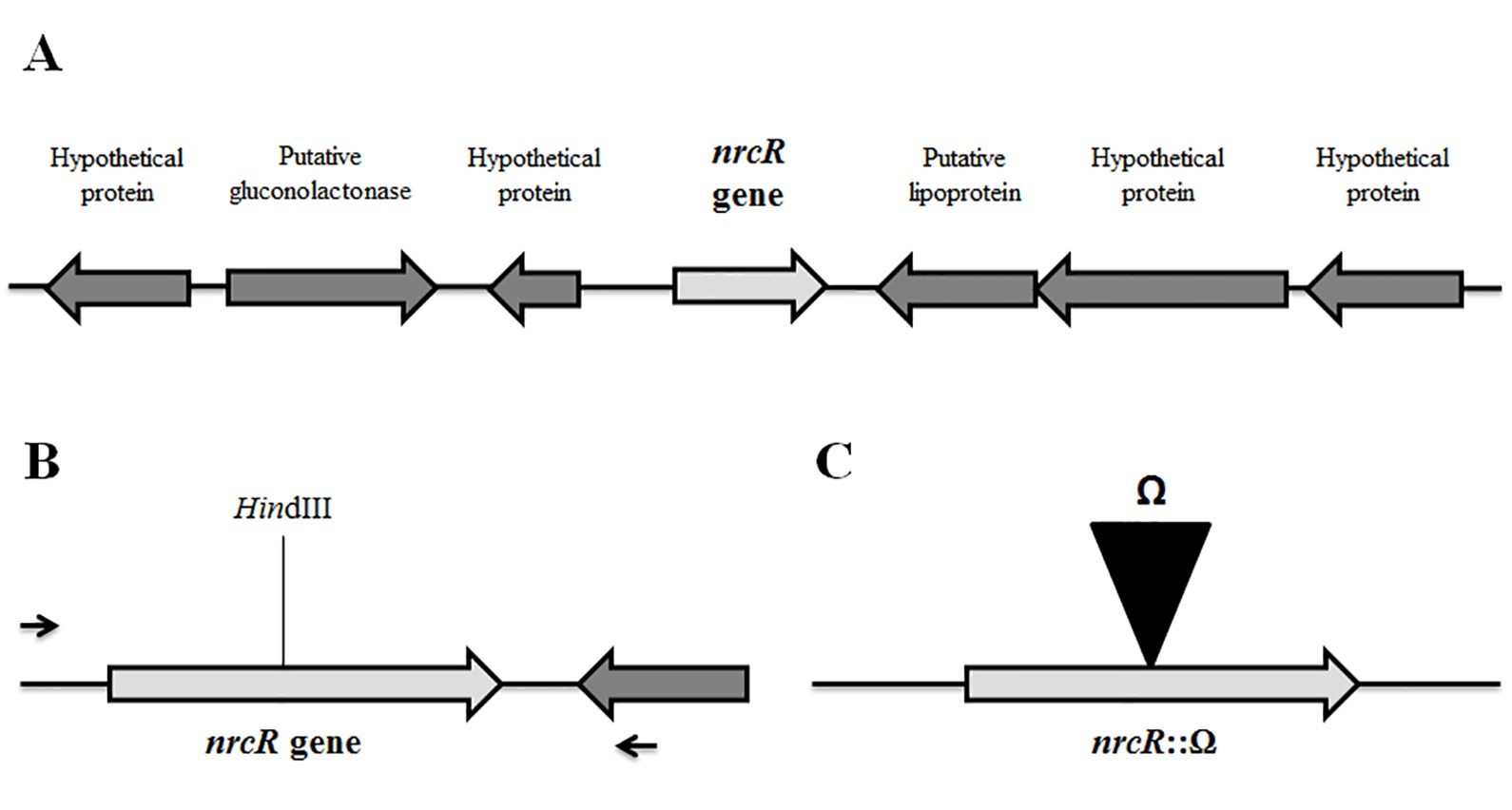

Supplement: S1 Fig — (A) Gene neighborhood of nrcR gene in the pRtrCIAT899c of R. tropici CIAT 899 genome. (B) HindIII endonuclease point and primers nolR-like-F and nolR-like-R location (C) Mutant interposon insertion. (TIF) [file pone.0154029.s001.tif]

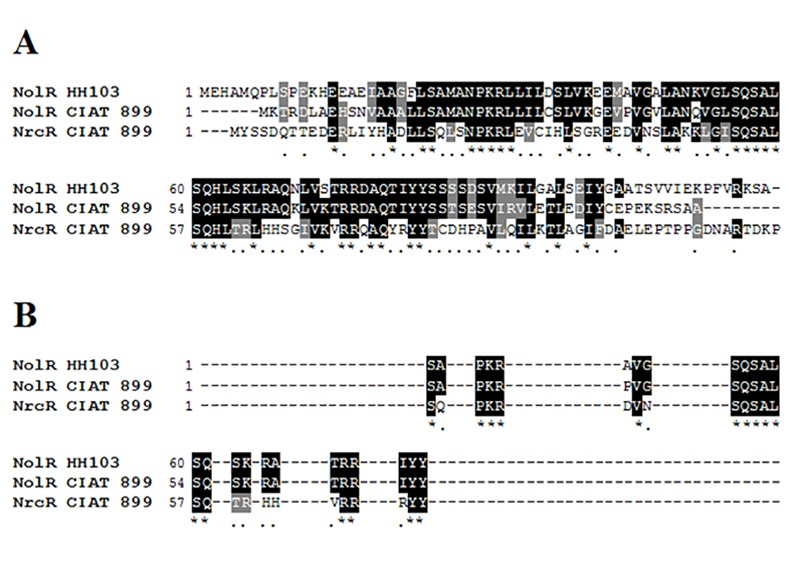

Supplement: S2 Fig — Black and gray boxes indicate identical and similar amino acids, respectively. (A) Complete sequence alignment of NolR of S. fredii HH103, and NolR, ArsR and NrcR proteins of R. tropici CIAT 899. (B) Putative DNA-binding domain of NolR of S. fredii HH103, and NolR and NrcR of R. tropici CIAT 899. (TIF) [file pone.0154029.s002.tif]

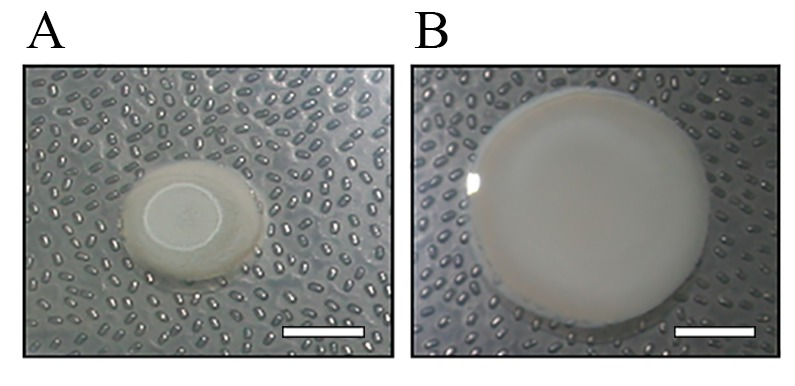

Supplement: S3 Fig — (A) CIAT 899. (B) nrcR::Ω mutant. (TIF) [file pone.0154029.s003.tif]
