# Supplementary material for: NrcR, a New Transcriptional Regulator of Rhizobium tropici CIAT 899 Involved in the Legume Root-Nodule Symbiosis
Source: PLoS One. 2016 Apr 20;11(4):e0154029. doi: 10.1371/journal.pone.0154029 (PMC4838322; doi:10.1371/journal.pone.0154029)
Supplement: S1 Table — (PDF) [file pone.0154029.s004.pdf]

S1 Table. Identity of the NrcR protein from *Rhizobium tropici* CIAT 899 with different ArsR and NolR proteins found in other *Rhizobium* and *Agrobacterium* strains.

Strain	Protein and family	Identity	Query cover	NCBI Accession
Rhizobium tropici CIAT 899	ArsR family protein (NrcR)	100%	100%	WP_015343102.1
Rhizobium tropici PTD1	Undescribed ArsR family protein	91%	89%	WP_052227882.1
Agrobacterium rhizogenes OK036	Undescribed ArsR family protein	80%	100%	WP_034484510.1
Rhizobium sp. OK494	Undescribed ArsR family protein	84%	90%	WP_051964083.1
Rhizobium leguminosarum YR374	Undescribed ArsR family protein	84%	90%	WP_052212628.1
Agrobacterium rhizogenes YR147	Undescribed ArsR family protein	75%	100%	WP_051976725.1
Rhizobium tropici YR635	Undescribed ArsR family protein	79%	92%	WP_047639624.1
Multispecies *Rhizobium*/*Agrobacterium* group	Undescribed ArsR family protein	70%	75%	WP_034515914.1
Rhizobium sp. YR060	Undescribed ArsR family protein	69%	75%	WP_037219691.1
Rhizobium freirei PRF 81	Undescribed ArsR family protein	70%	70%	WP_004124074.1
Sinorhizobium fredii HH103	NolR protein	45%	72%	AAO27299.1
Sinorhizobium meliloti SM11	NolR protein	48%	75%	AEH79805.1
Rhizobium etli sv. *phaseoli* IE4803	NolR protein	41%	82%	AJC82776
Rhizobium tropici CIAT 899	NolR protein	46%	70%	AGB71986

1
